# Author Correction: The nucleotide pGpp acts as a third alarmone in *Bacillus*, with functions distinct from those of (p)ppGpp

**DOI:** 10.1038/s41467-021-24209-2

**Published:** 2021-06-16

**Authors:** Jin Yang, Brent W. Anderson, Asan Turdiev, Husan Turdiev, David M. Stevenson, Daniel Amador-Noguez, Vincent T. Lee, Jue D. Wang

**Affiliations:** 1 Department of Bacteriology, University of Wisconsin, Madison, WI USA; 2 Department of Cell Biology and Molecular Genetics, University of Maryland, College Park, MD USA

**Keywords:** Nucleotide-binding proteins, Stress signalling, Bacterial physiology

Correction to: *Nature Communications* 10.1038/s41467-020-19166-1, published online 23 October 2020.

The original version of this Article contained an error in Fig. 1d, in which the schematic arrow related to HflX was incorrectly placed between 100S and 50S ribosomes, rather than between 100S and 70S ribosomes.

In addition, the original version of this Article contained an error in the title, which was previously incorrectly given as ‘The nucleotide pGpp acts as a third alarmone in *Bacillus*, with functions distinct from those of (p) ppGpp’. The correct version states ‘(p)ppGpp’ in place of ‘(p) ppGpp’.

Both errors have been corrected in both the PDF and HTML versions of the Article.

