# Supplementary material for: The nucleotide pGpp acts as a third alarmone in Bacillus, with functions distinct from those of (p)ppGpp
Source: Nat Commun. 2020 Oct 23;11:5388. doi: 10.1038/s41467-020-19166-1 (PMC7584652; doi:10.1038/s41467-020-19166-1)
Supplement: Supplementary file 1 — Supplementary Information [file 41467_2020_19166_MOESM1_ESM.pdf]

Fig. S1

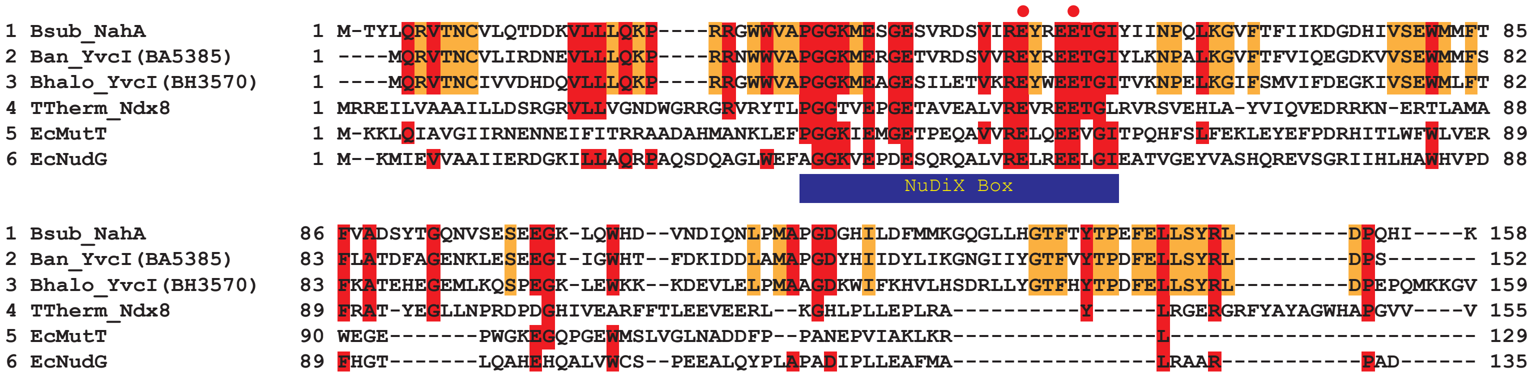

Figure S1. Protein sequence alignment of NahA (YvcI) homologs in different species.

Multiple sequence alignment of YvcI homologs from *B. subtilis* (Bsub), *B. anthracis* (Ban), *B. halodurans* (Bhalo), *Thermus thermophilus* (TTherm) and *E. coli* (Ec). Alignment was obtained with MAFFT. Red shading indicates identical residues among *Bacillus* and other species, and orange shading indicates identical residues only in *Bacillus* species. The *Bacillus halodurans* YvcI structure (PDB ID: 3FK9) shows its conserved structure as a NuDiX hydrolase. Red dots label the key residues for catalytic activity. Numbers on the sides of the sequences indicate the residue numbers in the corresponding proteins.

Fig. S2

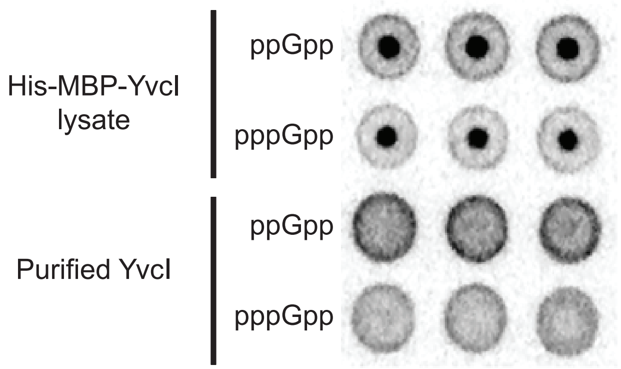

Figure S2. Cell lysate containing YvcI, but not purified YvcI, binds (p)ppGpp.
DRaCALA of cell lysate containing His-MBP-tagged *B. subtilis* YvcI (NahA) and purified His-MBP-tagged *B. subtilis* YvcI (NahA) with 5'-α-^{32}P-labeled (p)ppGpp.

Fig. S3

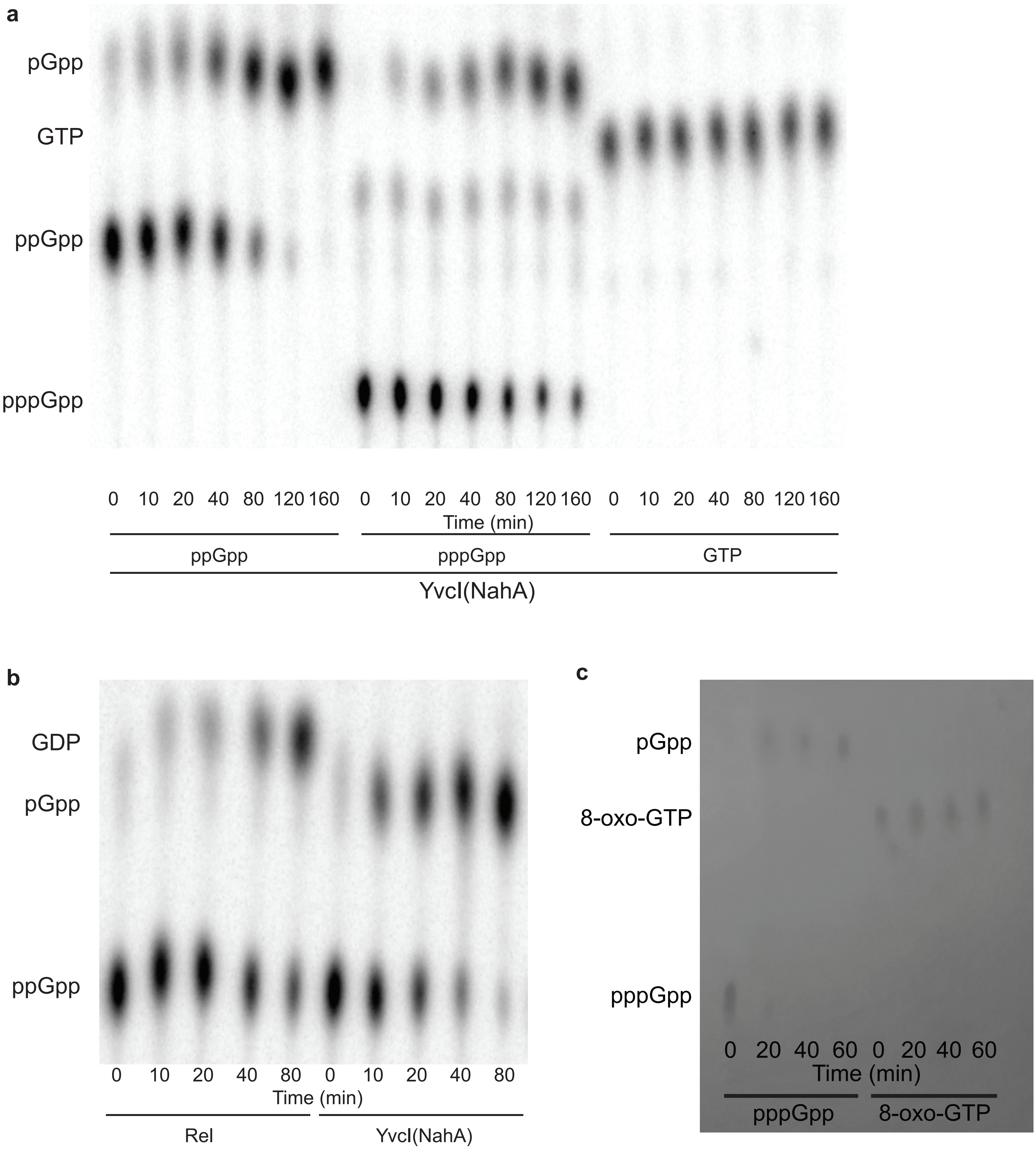

Figure S3. NahA hydrolyzes (p)ppGpp *in vitro*.
a) TLC analysis of pppGpp and ppGpp hydrolysis by NahA over time. The same assay was also performed using GTP. **b)** TLC analysis of ppGpp hydrolysis by Rel and NahA over time. Substrate and product identities are labeled on the left side of the figure. **c)** pppGpp and 8-oxo-GTP hydrolysis reaction, separated by TLC and visualized by UV. 1 mM pppGpp and 8-oxo-GTP were incubated with 200 nM *B. subtilis* NahA at 37°C. Samples were collected at time points denoted in the figure and analyzed by TLC. TLC plate was imaged with UV at wavelength of 254 nm.

Fig. S4

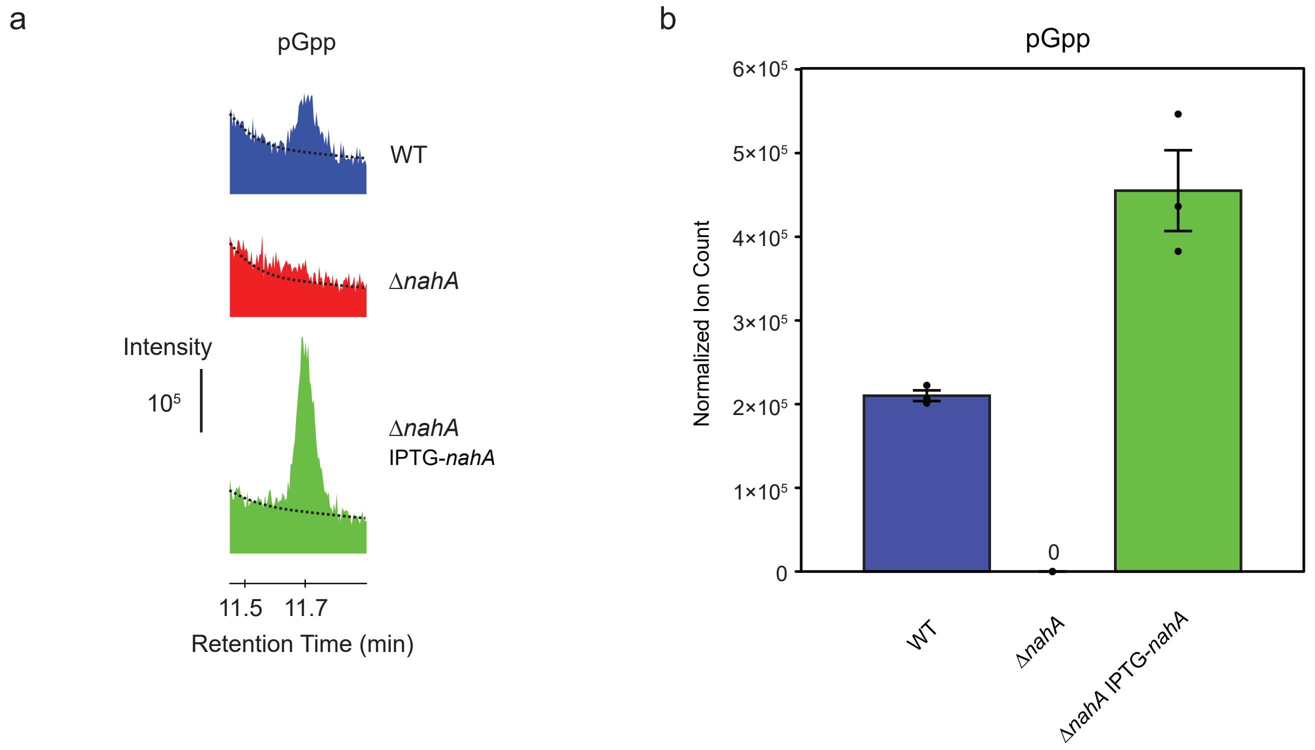

Figure S4. *nahA* complementation rescues pGpp production *in vivo*.
a) Intensity profile of pGpp from LC-MS measurement of mid log phase wild type, Δ*nahA* and *nahA* complementation strain (Δ*nahA* IPTG-*nahA*). x-axis: retention time in LC-MS; y-axis: ion counts. For each strain, a representative curve was shown. Dashed lines are the shoulders of the GTP signal peak. **b)** Normalized ion count (ion count per OD_{600nm} per unit volume of the culture) of corresponding pGpp levels, obtained as the average of 3 biological replicates in a). Error bars represent standard error of the mean.

Fig. S5

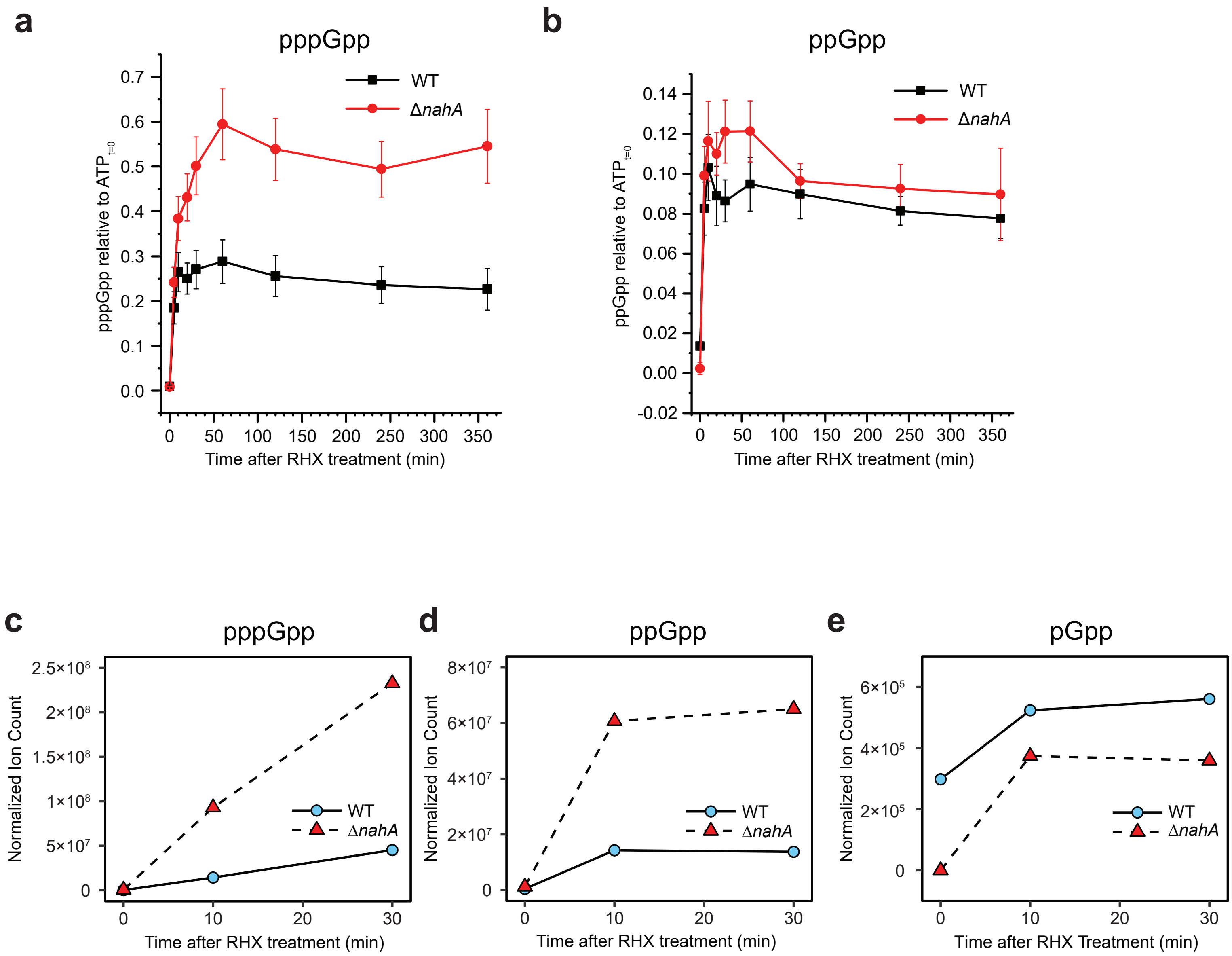

Figure S5. *nahA* mutant displayed higher (p)ppGpp and lower pGpp *in vivo* in *B. subtilis* in a time course of amino acid starvation.

a-b) pppGpp (a) and ppGpp (b) levels in wild type and Δ*nahA B. subtilis* cells treated with the nonfunctional arginine analog RHX (arginine hydroxamate). Nucleotide levels were assayed via TLC and are normalized to the ATP level at time zero ($ATP_{t=0}$). Error bars represent standard errors of mean of three independent experiments. **c-e)** LC-MS analyses of pppGpp (c), ppGpp (d) and pGpp (e) of wild type and Δ*nahA* in a time course of RHX treatment. Normalized ion count is ion count per OD_{600nm} per unit volume of the culture. The average shown in figure represents two biological replicates.

Fig. S6

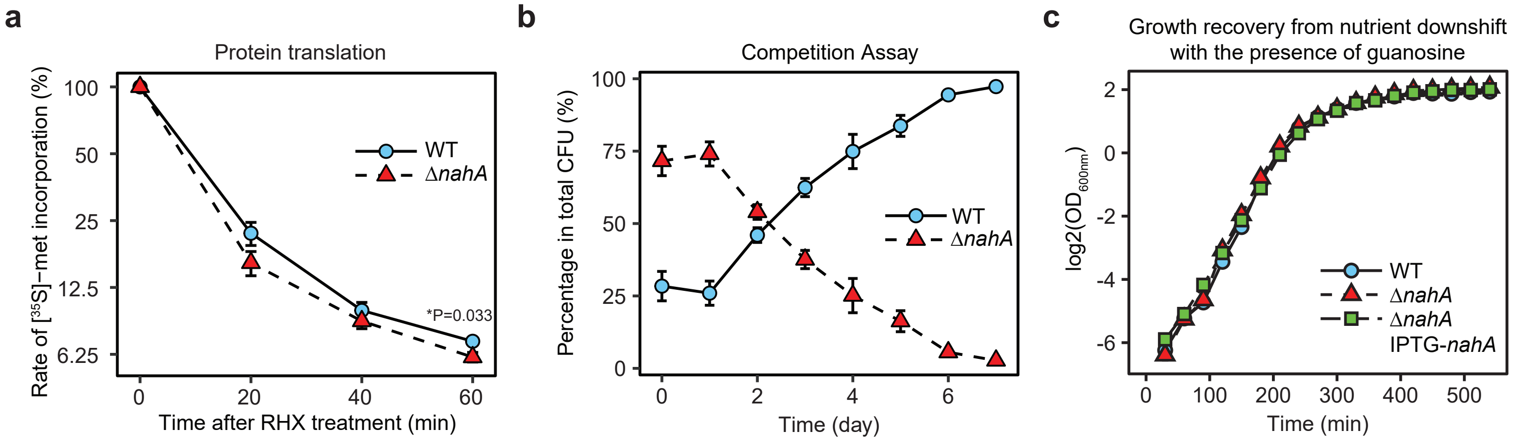

Figure S6. Phenotypical effects of *nahA* deletion.

a) Protein translation rate of wild type and Δ*nahA* cells in a time course of arginine hydroxamate (RHX) treatment. Translation rate was measured by a two minutes pulse of [^{35}S]-methionine incorporation into TCA-precipitable fraction. Error bars represent standard errors of the mean from three replicates. Asterisks indicate statistical significance of differences (* $P \leq 0.05$, two-tailed two-sample equal-variance Student's *t* test). **b)** Percentage of Δ*nahA* and wild type strains obtained from a 7-day serial-dilution competition assay. Error bars represent standard errors of the mean from 3 repeats. **c)** Log phase cultures of wild type, Δ*nahA* and *nahA* complementation (Δ*nahA* IPTG-*nahA*) strains in rich media with 0.5 mM guanosine were firstly downshifted in minimum media without amino acids for 10 min, and then diluted in rich media with 0.5 mM guanosine for outgrowth. Error bars in represent standard errors from 9 replicates.

Supplementary Table 1. K_d ± SD of GTPases to pppGpp, ppGpp and pGpp. ND: not detected.

	pppGpp	ppGpp	pGpp
HflX	5 ± 1 μM	3.0 ± 0.6 μM	ND
Obg	48 ± 7 μM	6 ± 2 μM	ND
EF-G	296 ± 50 μM	60 ± 8 μM	ND
