# Supplementary material for: The nucleotide pGpp acts as a third alarmone in Bacillus, with functions distinct from those of (p)ppGpp
Source: Nat Commun. 2020 Oct 23;11:5388. doi: 10.1038/s41467-020-19166-1 (PMC7584652; doi:10.1038/s41467-020-19166-1)
Supplement: Supplementary file 3 — Description of Additional Supplementary Files [file 41467_2020_19166_MOESM3_ESM.pdf]

Description of Additional Supplementary Files

File Name: Supplementary Data 1

Description: Summary of pGpp, ppGpp and pppGpp binding targets. Fractions of different radiolabel binding under different conditions are listed. Z-score is calculated by the formula that Z-score = (logit(x)-logit(median))/SD(logit(x)), where logit(x) = $\log_{10}(x/1-x)$, "x" is the fraction of radiolabel binding, "median" is the median of "x" on one plate, "SD(logit(x))" is the standard deviation of "logit(x)" on one plate. Calculation of Z-scores ignores "x" that is below or equals zero.* The differences in the fraction of pppGpp binding of some proteins in different libraries might be due to the differential expression levels from His-tagged and HisMBP-tagged vectors. Different solubility of His-tagged and HisMBP-tagged proteins (MBP tends to increase the solubility of tagged protein) may also contributes to this difference. NT: not tested; NA: not available due to 0% radiolabel binding.

File Name: Supplementary Data 2

Description: Normalized ion counts of metabolites measured by LC-MS (Raw data of Figure 4a).
